# Identifying emotional components of event-related potentials in the brain functioning of individuals with contamination obsessions and comparison with healthy control group

**DOI:** 10.3389/fpsyg.2023.1240493

**Published:** 2023-11-16

**Authors:** Seyed Hamid Seyednezhad Golkhatmi, Behrooz Dolatshahi, Masoud Nosratabadi, Shima Shakiba, Seyed Alireza Sadjadi

**Affiliations:** ^1^Department of Clinical Psychology, University of Social Welfare and Rehabilitation Sciences, Tehran, Iran; ^2^Psychiatry and Behavioral Sciences Research Center, Mashhad University of Medical Sciences, Mashhad, Iran

**Keywords:** OCD, N2, P3, contamination, ERP, EEG

## Abstract

The present study aimed to examine the emotional components of event-related potentials (ERPs) in individuals with contamination OCD and compare them with a healthy control group. A convenience sample of 45 participants was included, consisting of 30 individuals diagnosed with contamination-type OCD and 15 individuals in a healthy control group. Both groups participated in an ERP study where they encountered a computer-based task presenting both contamination and neutral pictures, while their brain activity was recorded. The data were analyzed using repeated measures analysis of variance (RANOVA) with SPSS-24 and Matlab software. Findings suggest that in P3 amplitude, only individuals with OCD exhibited a larger positive amplitude (*p* < 0.05) in response to contaminated pictures compared to neutral pictures and in N2 amplitude, only individuals with OCD exhibited a larger negative amplitude (*p* < 0.05) in response to contaminated pictures compared to neutral pictures in the central vertex (Fz). These findings hold promising implications for the development of more targeted and effective treatments for contamination OCD, emphasizing the importance of emotion-oriented approaches to address the unique neural patterns observed in the frontal vertex.

## Introduction

Obsessive-compulsive disorder (OCD) is characterized by the presence of intrusive and unwanted thoughts, images, or ideas that involuntarily enter one’s consciousness (known as obsessions). Individuals with OCD often engage in repetitive behaviors such as checking or mental rituals like praying in an attempt to neutralize these obsessions (known as compulsions). OCD is recognized as one of the most debilitating psychiatric disorders, affecting people across cultures, with a consistent prevalence of nearly 2% throughout their lives. The onset of OCD typically occurs during adolescence, and it is more prevalent among women, with a ratio of 1.5 to 1 compared to men (American Psychiatric Association, 2013). The two prominent clinical features of OCD include rituals and avoidance behaviors which manifest in both behavioral and mental forms. Among the rituals experienced by individuals with OCD washing and cleaning are the most prevalent which are carried out to eradicate perceived “contamination” originating from specific sources (e.g., microbes or chemicals). Individuals suffering from OCD often endure intense contamination anxiety, a prevalent and conspicuous aspect of the disorder. This anxiety is commonly manifested through intrusive thoughts or vivid mental imagery, both of which are closely associated with potential sources of contamination. These sources can range from commonplace substances like dirt, and bodily fluids, to mold, further intensifying their distress (Franklin and Foa, 2011). Contamination Obsession can be described as a strong and persistent sensation of being tainted, soiled, infected, or at risk due to contact, whether direct or indirect, with something or someone perceived as unclean, impure, infectious, or harmful (Rachman, 2006). This fear is commonly associated with OCD, where obsessions often revolve around concerns related to germs, disease, or overall cleanliness, while compulsions frequently manifest as repetitive washing rituals (Rachman, 2004, 2006). Contamination-related OCD is notably prevalent among the various clinical presentations of OCD (Ruscio et al., 2010), affecting up to 46% of individuals with the disorder, leading to significant fears of contamination and accompanying compulsive washing behaviors (Jalal et al., 2020). This subtype of OCD exhibits distinct characteristics, including specific patterns of neural activity such as notable consistent elevations in bilateral amygdala activation (Lindquist et al., 2012), and obsessive beliefs such as an exaggerated perception of threat (Tolin et al., 2008). Patients often grapple with apprehensions related to the repercussions of contamination, both in a general sense and within specific contexts. These concerns encompass fears of falling ill, succumbing to illness, or inadvertently causing harm or sickness to others (Clark and Simos, 2013).

Understanding the pathophysiology of OCD remains a challenge, limiting the efficacy of current treatments and hindering the development of more effective interventions (Taylor et al., 2012; Fineberg et al., 2020). Therefore, further research is crucial to uncover the underlying mechanisms of OCD. While cognitive impairments have been extensively studied in OCD, the role of emotional factors in the development of obsessions has received less attention. However, there is substantial evidence supporting the presence of emotional dysregulation in OCD (Kornreich et al., 2001). Individuals with OCD often exhibit heightened emotional arousal and difficulties in executive control, affecting various regions of the brain. This emotional dysregulation also influences the response to stress and the functioning of neuropeptides within the emotional system (Alonso et al., 2021; Goldstein-Piekarski and Williams, 2021).

To investigate emotional regulation impairments in OCD, researchers have utilized event-related potentials (ERPs), which offer several advantages, including direct measurement of neural activity, high temporal resolution, cost-effectiveness, and suitability for a wide range of participants (Perera et al., 2023). ERPs represent voltage changes identified in EEG that occur in response to timed stimuli, providing valuable insights into the neural processes involved in emotional regulation (Coles and Rugg, 1995). Several ERP components have been identified that show larger amplitudes in response to emotional stimuli compared to neutral stimuli, with a specific focus on the P3 and N2 components (Hajcak and Foti, 2020). P3 is a positive ERP component that is typically observed over central or parietal regions and is enhanced by the presentation of stimuli in both non-emotional (e.g., target status or rarity in an odd-ball paradigm; Hajcak and Foti, 2020) and emotional contexts (Weinberg et al., 2012). It is believed to reflect common emotional and motivational responses (Hajcak and Foti, 2008; Bradley, 2009). More specifically, in tasks involving emotional picture viewing, the P3 component typically reaches its peak amplitude between 300 and 600 milliseconds after stimulus onset and exhibits maximal activity in central and parietal scalp locations. On the other hand, the N2 component is a negative deflection in brainwave activity occurring around 200 milliseconds after stimulus presentation (Kloft et al., 2019). In individuals with OCD, the N2 component has been found to be altered in response to emotional stimuli, particularly stimuli associated with anxiety or threat (Cavanagh and Shackman, 2015). Overall, the N2 and P3 components of ERP can provide valuable insights into the neural mechanisms underlying emotional processing in individuals with OCD. By measuring changes in N2 and P3 amplitudes in response to emotional stimuli, researchers may be able to identify neural markers that can be utilized to develop more targeted treatments for OCD and enhance our understanding of the disorder (Cavanagh and Shackman, 2015).

Research literature has yielded conflicting findings regarding the amplitude and localization of N2 and P3 components in OCD. For instance, studies have reported varying degrees of N2 amplitude in individuals with OCD compared to healthy individuals (Riesel et al., 2017). Inconsistent findings have also been reported for P3, with some studies indicating higher ranges (Ischebeck et al., 2011; Andreou et al., 2013) and others suggesting lower ranges (Yamamuro et al., 2015) in OCD. Additionally, conflicting results have emerged regarding the role of N2 amplitude in OCD, with a few studies investigating response generation and inhibition processes using ERP. For instance, Ruchsow et al. (2007) reported an increase in N2 ranges, whereas Kim et al. (2007) reported a decrease in N2 ranges in OCD. Furthermore, Herrmann et al. (2003) found a normal N2 range but a shorter N2 delay in OCD patients compared to the healthy control group. The existing research literature presents conflicting findings regarding the amplitude and localization of N2 and P3 components in OCD, with a particular emphasis on the contamination subtype. While numerous studies have reported differing N2 amplitude levels in individuals with OCD compared to healthy controls (Riesel et al., 2017), these investigations predominantly concentrated on the general OCD population, lacking a comprehensive examination of contamination-specific aspects. It is possible that the inconsistent findings can be attributed to the lack of consideration of OCD subtypes. In the meantime, it’s crucial to acknowledge that only a limited number of studies have encompassed and analyzed individuals with contamination-type OCD, and it should be noted that in these studies, not all participants exhibited an obsession with the specific type of contamination. For instance, in Wang et al.’s (2020) study, individuals with high contamination fears exhibited heightened P1 amplitude, indicative of increased vigilance to threats potentially influenced by the amygdala. In another study by Zhang et al. (2017) a comprehensive examination of the contamination subtype of OCD was conducted, exploring the intricate aspects of ERP, including N2 and P3 components, within the context of contamination-related OCD. The outcomes of this investigation underscored the significant roles of these components in both cognitive and emotional processing domains. Meanwhile, further studies are needed to provide a more comprehensive understanding of the amplitude and localization of N2 and P3 components in the contamination OCD subtype.

This study aims to address the existing knowledge gap regarding OCD with contamination features by investigating its underlying neural mechanisms using event-related electroencephalography (EEG) potentials. By introducing ERPs within the context of contamination-related OCD, we aim to bridge the gap between behavioral and neural research on the emotional dimensions of this condition. Our specific objective is to extract relevant potentials through tasks designed to elicit contamination-related emotional responses, facilitating a comparative analysis of neural processes and brain activity between individuals diagnosed with contamination-type OCD and a healthy control group. Previous studies consistently highlight differences in EEG measurements between individuals with OCD and healthy controls (Perera et al., 2019), emphasizing the importance of exploring these neural markers, particularly within the domain of contamination-related OCD symptoms.

## Method

### Participants and procedure

A total of 30 patients with OCD (16 females and 14 males) and 15 individuals in the healthy control groups (8 females and 7 males) were recruited for the present study. The patients were selected from the outpatient unit of the Department of Psychiatry at Mashhad University of Medical Sciences (Ibn Sina Hospital). All patients included in the study underwent a thorough diagnosis of OCD using the structured clinical interview for DSM-5 conducted by trained psychiatrists. Furthermore, participants rated 10 items from the contamination obsession subscale of the Padua Test (see measures section) and were required to score 8 or higher to meet the criteria of contamination-related OCD (Broderick et al., 2013). Healthy comparison participants were recruited through community advertisements and from the patients’ companions. It is worth noting that among the healthy control group, three out of fifteen participants were companions of the OCD patients, but these companions were not first-degree relatives of the patients but rather individuals from the patients’ social circles who volunteered to participate as part of the healthy control group. This approach was implemented to minimize potential bias associated with close genetic relationships. Both groups were carefully matched in terms of age and education level. The age range for all participants was between 20 and 50 years, and they reported no history of head injury or neurological illness. Patients with lifetime diagnoses of psychotic or substance-related disorders were excluded from the study. The healthy control group had additional exclusion criteria, including current or past psychological treatment, psychoactive medication use, and any current or past psychiatric disorder. These criteria were assessed using the Structured Clinical Interview for DSM-5 (SCID).

All participants were provided with written and verbal information about the study, and they subsequently gave their written informed consent. Then, participants underwent an ERP during which they were presented with a computer-based task that included both contamination and neutral pictures. Their brain activity was recorded while they were exposed to the contamination pictures. The main objective of this test was to identify the specific neural processes associated with contamination obsessions by comparing the brain regions activated by provocative cues (disgust pictures) and neutral stimuli (landscapes, everyday objects) between the group with obsessive symptoms and the group without such symptoms. The study procedures adhered to the ethical guidelines of the University of Social Welfare and Rehabilitation Sciences and were approved by the university’s ethics committee (Code Number = IR.USWR.REC.1398.018).

### Measures

#### Padua inventory (modified by Washington State University)

The Padua Inventory is a widely used self-report questionnaire consisting of 60 items rated on a five-point scale (0 = not at all to 4 = very much). The measure was initially developed by Sanavio (1988) to assess the severity of problems associated with thoughts, behaviors, and compulsions. However, subsequent revisions were made to address the high correlation with anxiety and difficulties in differentiating between anxiety and obsessive thoughts. The revised version, known as the Padua Inventory (revised version from the University of Washington), was developed by Burns et al. (1996) and contains 39 items. The questionnaire encompasses various factors, including contamination obsessions, cleaning compulsions, ordering and arranging compulsions, hoarding compulsions, obsessive thoughts of harm to oneself and others, obsessive thoughts of violence, compulsive checking of damage to oneself and others, and compulsive stealing. Several studies have demonstrated high internal consistency for both the total score and subscales of the Padua Inventory (with Cronbach’s alpha values exceeding 0.80). In Iran, Shams et al. (2019) examined the validity and reliability of the Padua Inventory among 438 individuals aged 18–44. The results showed high internal consistency (Cronbach’s alpha = 0.92), strong split-half reliability (*r* = 0.95), and good test–retest reliability (*r* = 0.77). In this study, we administered 10 items of the Padua Inventory which assesses contamination obsessions.

#### The international affective picture system

The IAPS (Lang et al., 2008) is a standardized set of images used in psychological research to elicit emotional responses from participants. While the primary focus of the IAPS is to elicit emotional responses, it also includes images that are considered neutral or emotionally ambiguous. These neutral images serve as a baseline for comparison and allow researchers to differentiate between emotional and non-emotional responses. For the aims of this study, we used 50 neutral pictures from the IAPS. In the context of this study, it is worth noting that Saremi et al. also employed these neutral pictures from the IAPS in their research conducted in Iran (Saremi et al., 2016).

#### The Berlin obsessive-compulsive disorder picture set

The BOCD-PS, a standardized set of images specifically developed for research and assessment in OCD (Simon et al., 2012; Basel et al., 2023), has undergone extensive validation in both OCD and non-clinical populations, making it suitable for use with both clinical and non-clinical groups. This image set was created based on the main symptoms of OCD as outlined in the fourth revised edition of the Diagnostic and Statistical Manual of Mental Disorders (DSM-IV). The BOCD-PS consists of seven subscales representing different symptom dimensions of OCD: Contamination (50 pictures), checking (25 pictures), counting (25 pictures), aggression (25 pictures), order and symmetry (25 pictures), hoarding (25 pictures), and religious (25 pictures). Each subscale contains a specific set of images designed to elicit responses related to the corresponding symptom dimension. To assess participants’ reactions to the images, three grading scales were utilized: anxiety related to OCD (ranging from “not at all” = 1 to “severe” = 9), arousal (ranging from “low” = 1 to “severe” = 9), and valence (ranging from “low” = 1 to “severe” = 9). These scales provide quantitative measures of anxiety, arousal, and emotional valence associated with the presented images. The psychometric properties of the BOCD-PS have been examined in clinical samples and healthy control groups, including the assessment of Cronbach’s alpha (*a* ≥ 0.96) for OCD-related anxiety, arousal, and negative valence (Saremi et al., 2016). In the present study, a subset of 50 contamination pictures from the BOCD-PS was selected. Additionally, a preliminary study was conducted to evaluate the Iranian version of the BOCD-PS, involving feedback from five clinical psychologists and psychiatrists with experience in treating OCD patients. Their input was obtained to assess content validity and determine the need for potential picture replacements, taking into consideration the cultural context of Iran (Saremi et al., 2016).

#### Structured clinical interview for DSM-IV

The Structured Clinical Interview for DSM-5 Disorders is a standardized interview used for clinical and research purposes to assess major psychiatric disorders according to DSM-5 definitions and criteria (Osório et al., 2019). It was originally developed by First et al. (1997). In Iran, Mohammadkhani et al. (2020) showed that the Persian SCID-5 is a valid and reliable tool. The internal consistency of the SCID-5 was found to be high, with Cronbach’s alpha values ranging from 0.95 to 0.99 for all assessed disorders. Test–retest reliability, assessed after a two-week interval, ranged from 0.60 to 0.79, indicating moderate to substantial reliability. Additionally, kappa reliability coefficients ranged from 0.57 to 0.72. In the present study, the SCID-5 interview was utilized to evaluate and screen patients for major psychiatric disorders based on DSM-5 criteria.

### Computerized emotional task

For the design of this study, a total of 100 pictures were utilized, comprising 50 contamination-related pictures from the BOCD-PS and 50 neutral pictures from the IAPS. These contamination-related pictures were specifically chosen to elicit emotional responses in participants and are tailored to the context of OCD with contamination characteristics. These pictures encompassed various forms of contamination, including environmental pollution, bodily pollution, and body secretions, among others. All pictures were displayed on a 15-inch computer monitor with a specific resolution and pixelation for a duration of 4,000 milliseconds. The presentation order of the pictures was pseudo-randomized using the Encephalan software. This software utilizes a sophisticated randomization algorithm that considers factors such as stimulus characteristics and experimental conditions to generate a presentation sequence that appears random to participants. By adhering to the predefined experimental parameters, this approach aimed to reduce potential order effects and minimize unintended influences on participant responses. During the presentation, both neutral and contamination-related pictures were preceded by an exclamation mark (!) displayed on the monitor for 500 milliseconds, followed by a 700-millisecond break. Subsequently, the picture itself appeared on the screen for 1,000 milliseconds. Participants were instructed to pay attention to this warning sign. Immediately after the warning sign, with a slight delay of 50 milliseconds, the neutral or contamination-related picture was displayed for 4,000 milliseconds. To prepare the participant for the subsequent picture, a white screen appeared for 1,500 milliseconds, with a delay of 500 milliseconds. Prior to the actual experiment, two neutral pictures were presented as a practice to familiarize the participants with the task.

#### Analysis, reduction, and recording of electroencephalography

In this study, EEG recordings were conducted using a standard EEGR device manufactured by Medicom Russia. The international standard 20–10 system was employed, utilizing 19 channels positioned in accordance with the average of both ears. To ensure optimal recording quality, the impedance of all electrodes was carefully maintained below 10 kilohms. During the recording session, a sampling rate of 500 Hz was employed, and the electrode band-pass filter was set between 0.05 and 70 Hz. These parameters ensured the capture of relevant neural activity within the desired frequency range. To improve the signal quality, artifacts associated with eye movements and blinking were removed during the offline data analysis phase. This artifact removal process was performed using the Gratton and Coles algorithm (Gratton et al., 1983), a well-established method in the field of EEG analysis.

The EEG activity was further filtered using a 0.15–50 Hz band-pass filter. The data was divided into time segments of 1700 milliseconds each, consisting of 100 milliseconds before and 1,600 milliseconds after stimulus presentation. Time segments with amplitudes exceeding 100 millivolts were excluded from the analysis. The mean amplitude of the 100 milliseconds preceding the stimulus presentation was considered the baseline. After baseline correction, average event-related potentials (ERPs) were separately calculated for neutral and contamination-related pictures. To streamline the analysis and reduce the volume of data, instead of examining individual electrodes, the brain was divided into regions using a brain segmentation method (Dien and Santuzzi, 2005). The following electrode assignments were made: F4 for the right frontal cortex, F3 for the left frontal cortex, C4 for the right motor cortex, C3 for the left motor cortex, P4 for the right parietal cortex, P3 for the left parietal cortex, and Fz, Cz, and Pz for the central vertex (Cz). The qualitative analysis of ERPs was performed using the Matlab software.

For statistical analyses, SPSS version 24.0 was employed. Descriptive analyses were initially conducted, followed by repeated measures analysis of variance (RANOVA) for different regions, namely Fz, Cz, and Pz, as well as for pairs of regions (P3, P4), (C3, C4), and (F3, F4). Considering the significance of exploring the left and right hemispheres within the frontal, central, and parietal regions, as well as the central vertex of the head, four separate RANOVA analyses were performed to investigate ERP components in these areas. It should be noted that distinct analyses were conducted for each ERP component. In terms of the RANOVA, the assumptions of covariance matrix sphericity should be met, though whenever this assumption was violated in any of the analyses, the Greenhouse–Geisser correction was applied to adjust the degrees of freedom for *F* values. In the RANOVA equation, the group factor (obsessive-compulsive, normal) was included as a between-group factor, while the cue (contaminated, neutral) and electrode for example (amplitude values of Fz, Cz, and Pz) were included as within-group factors to examine differences in various ERP components within the central vertex of the head, encompassing the frontal, parietal, and central regions. Partial eta squared (*η_p_*^2^) values of 0.01, 0.06, and 0.14 were interpreted as small, medium, and large effects, respectively.

## Results

### Demographic statistics

Table 1 displays the descriptive statistics for the two groups, as well as the results of the *t*-tests assessing the differences between them. The *t*-test analysis revealed that there was no significant difference in age between the groups [*t* (43) = 0.61, *p* = 0.29]. Additionally, the gender distribution between the groups showed no significant difference [*χ*^2^(2) = 0.10, *p* = 0.75].

**Table 1:** Descriptive indices related to the age of subjects in the obsession and normal groups.

Group	N	Mean	Standard deviation
Obsession	30	31.25	6.12
Normal	15	28.62	4.28

### Group differences

The RANOVA was conducted to explore differences in various ERP components within the central vertex of the head, including the frontal, central, and parietal regions (Table 2). The between-group factor included the variable of group (obsessive-compulsive, healthy controls), while the within-group factors included the cue (contamination, neutral) and electrode (Fz, Cz, and Pz). As we focus on measuring contamination effects, our RANOVA results exclusively highlight significant effects related to the cue across various components (Table 3).

**Table 2:** Descriptive indices of ERP components for contamination and neutral cues in two groups of individuals with contamination OCD and healthy control.

Component		P2		N2		P3		ELPP	
Electrode	Group	Mean		Mean		Mean		Mean	
		Contamination	Neutral	Contamination	Neutral	Contamination	Neutral	Contamination	Neutral
Fz	Obsession	2.85	2.55	−3.11	−0.47	4.24	1.99	−0.62	−0.56
	Normal	2.23	2.12	−0.93	−0.39	1.67	1.24	0.84	0.12
Cz	Obsession	2.96	2.81	−2.61	−1.99	3.76	3.10	−0.59	−0.46
	Normal	2.75	2.69	−1.49	−1.76	2.78	2.68	−0.07	−0.08
Pz	Obsession	3.00	2.89	−2.23	−1.16	3.33	3.01	−0.86	−0.87
	Normal	2.98	2.85	−1.51	−1.28	2.32	2.34	−0.06	−0.54
F3	Obsession	3.11	3.06	−1.61	−1.29	2.87	2.75	0.33	0.32
	Normal	2.79	2.57	−1.53	−1.33	2.46	2.11	−0.17	−0.14
F4	Obsession	3.57	3.48	−1.30	−0.88	3.17	3.13	0.32	0.31
	Normal	2.98	3.05	−1.36	−0.80	2.17	2.53	0.19	0.20
C3	Obsession	3.00	2.89	−1.50	−1.45	3.16	3.07	−0.38	−0.18
	Normal	2.78	2.79	−0.08	−0.50	2.64	2.67	−0.05	0.08
C4	Obsession	3.31	3.19	−1.28	−1.18	3.55	3.46	−0.37	0.09
	Normal	3.16	3.22	−1.16	−1.01	3.36	3.11	−0.02	0.09
P3	Obsession	3.68	3.36	−0.62	−0.46	4.40	3.75	−0.49	−0.36
	Normal	3.34	3.14	0.30	0.09	3.74	3.64	−0.45	0.09
P4	Obsession	4.22	4.18	−0.57	−0.49	4.37	4.27	−0.82	−0.66
	Normal	3.57	3.48	−0.21	−0.24	3.63	3.56	−0.48	−0.44

P2 = positive component 2; N2 = negative component 2; P3 = positive component 3; ELPP = early lateral positivity peaket; Fz = frontal midline; Cz = central midline; Pz = parietal midline; F3/F4 = left and right sides of the frontal lobe, respectively; C3/C4 = left and right sides of the central lobe, respectively; P3/P4 = left and right sides of the parietal lobe, respectively.

**Table 3:** Results of the analysis of variance with repeated measures (electrodes FZ, CZ, PZ).

Source	P2		N2		P3		ELPP	
	df	F	df	F	df	F	df	F
Electrode	42.2	2.73	42.2	3.38^*^	42.2	7.04^*^	42.2	3.11
Electrode × Group	42.2	0.38	42.2	0.72	42.2	2.84	42.2	0.24
Cue	43.2	0.85	43.2	7.21^*^	43.2	9.06^*^	43.2	2.23
Cue × Group	43.2	0.05	43.2	4.55^*^	43.2	4.64^*^	43.2	0.11
Electrode × Cue	42.2	0.70	42.2	3.23^*^	42.2	6.08^*^	42.2	0.38
Group × Cue × Electrode	42.2	0.01	42.2	0.80	42.2	2.52	42.2	0.15

P2 = positive component 2; N2 = negative component 2; P3 = positive component 3; ELPP = early lateral positivity peaket.

* *p* < 0.05.

The analysis revealed that two components, N2 and P3, were found to be significant in the central vertex of the brain. Table 3 demonstrates that the F statistic was statistically significant for the interaction effect of Cue × group (*F* = 4.55, *p* = 0.039, *η_p_*^2^ = 0.096), the interaction effect of Cue × Electhrod (*F* = 3.23, *p* = 0.049, *η_p_*^2^ = 0.133), and the cue effect (*F* = 7.21, *p* = 0.010, *η_p_*^2^ = 0.144) for the N2 component. Subsequent Bonferroni-corrected post-hoc *t*-tests were conducted to examine the nature of these differences. Only individuals with OCD exhibited a larger negative amplitude in response to contaminated pictures compared to neutral pictures in the Fz electrode vertex. Bonferroni-corrected post-hoc for the interaction effect of Cue × group (MD^1^ = −1.44, *p* = 0.039, *η_p_*^2^ = 0.096), the interaction effect of Cue × Electhrod (MD = −1.59, *p* = 0.015, *η_p_*^2^ = 0.133), and the cue effect (MD = −0.80, *p* = 0.010, *η_p_*^2^ = 0.144). No significant differences were observed between these points in the healthy control group.

**Figure 1:**
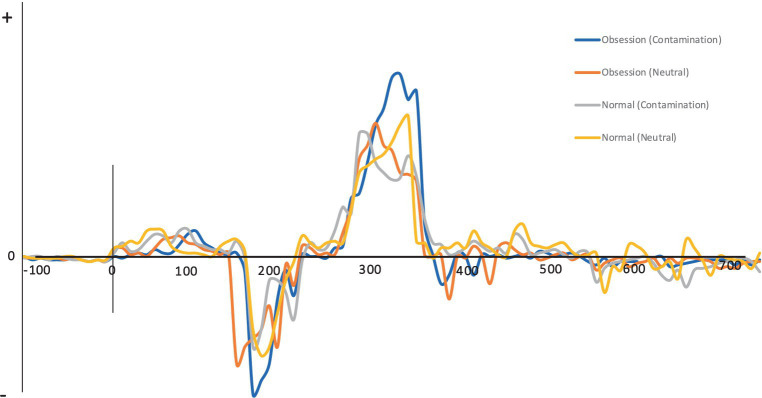
The status of groups in the P3 and N2 components.

Moreover, the F statistic was statistically significant for the interaction effect of Cue × group (*F* = 4.64, *p* = 0.037, *η_p_*^2^ = 0.097), the interaction effect of Cue × Electhrod (*F* = 6.08, *p* = 0.005, *η_p_*^2^ = 0.255) and the cue effect (*F* = 9.06, *p* = 0.004, *η_p_*^2^ = 0.174) for the P3 component. Subsequent Bonferroni-corrected post-hoc t-tests were conducted to examine the nature of these differences. Only individuals with OCD exhibited a larger positive amplitude in response to contaminated pictures compared to neutral pictures in the Fz electrode. Bonferroni-corrected post-hoc for the interaction effect of Cue × group (MD = 1.07, p = 0.037, *η_p_*^2^ = 0.097), the interaction effect of Cue × Electhrod (MD = 1.33, *p* = 0.125, *η_p_*^2^ = 0.108), and the Cue effect (MD = 0.62, *p* = 0.004, *η_p_*^2^ = 0.174). No significant differences were observed between contaminated and neutral pictures in the healthy control group. Figure 1 illustrates the status of groups in the P3 and N2 components.

Tables 4–6 present the F statistics for the main effects and interaction effects of ERP components in the frontal, central, and parietal regions of both hemispheres. None of these effects were found to be statistically significant, and no differences were observed between the OCD and healthy controls in these areas.

**Table 4:** Results of the analysis of variance with repeated measures (electrodes F3 and F4).

Source	P2		N2		P3		ELPP	
	df	F	df	F	df	F	df	F
Electrode	43.1	3.91	43.1	1.11	43.1	2.81	43.1	0.46
Electrode × Group	43.1	0.08	43.1	0.00	43.1	0.10	43.1	0.00
Cue	43.1	2.00	43.1	0.57	43.1	3.52	43.1	1.92
Cue × Group	43.1	0.00	43.1	0.00	43.1	0.10	43.1	0.005
Electrode × Cue	43.1	0.07	43.1	0.20	43.1	0.00	43.1	0.47
Group × Cue × Electrode	43.1	0.15	43.1	0.03	43.1	0.01	43.1	0.00

P2 = positive component 2; N2 = negative component 2; P3 = positive component 3; ELPP = early lateral positivity peaket.

**Table 5:** Results of analysis of variance with replicated data (electrodes C3 and C4).

Source	P2		N2		P3		ELPP	
	df	F	df	F	df	F	df	F
Electrode	43.1	2.52	43.1	0.61	43.1	3.21	43.1	0.19
Electrode × Group	43.1	0.00	43.1	0.31	43.1	0.06	43.1	0.11
Cue	43.1	0.20	43.1	3.71	43.1	1.54	43.1	0.87
Cue × Group	43.1	0.09	43.1	0.45	43.1	0.00	43.1	0.15
Electrode × Cue	43.1	0.04	43.1	2.58	43.1	0.13	43.1	0.06
Group × Cue × Electrode	43.1	0.00	43.1	0.08	43.1	0.06	43.1	0.08

P2 = positive component 2; N2 = negative component 2; P3 = positive component 3; ELPP = early lateral positivity peaket.

**Table 6:** Results of analysis of variance with replicated data (electrodes P3 and P4).

Source	P2		N2		P3		ELPP	
	df	F	df	F	df	F	df	F
Electrode	43.1	1.97	43.1	1.50	43.1	3.36	43.1	1.52
Electrode × Group	43.1	0.00	43.1	0.01	43.1	0.22	43.1	0.55
Cue	43.1	2.47	43.1	0.17	43.1	0.10	43.1	3.14
Group × Cue	43.1	0.10	43.1	0.01	43.1	0.38	43.1	0.44
Electrode × Cue	43.1	0.24	43.1	0.34	43.1	0.56	43.1	0.01
Group × Cue × Electrode	43.1	0.02	43.1	0.00	43.1	0.32	43.1	0.00

P2 = positive component 2; N2 = negative component 2; P3 = positive component 3; ELPP = early lateral positivity peaket.

## Discussion

As outlined in the Introduction section (Herrmann et al., 2003; Kim et al., 2007; Ruchsow et al., 2007; Ischebeck et al., 2011; Andreou et al., 2013; Yamamuro et al., 2015; Riesel et al., 2017; Zhang et al., 2017; Wang et al., 2020), the investigation of ERPs in OCD has produced a range of findings. It is crucial to highlight that many of these studies centered on the general OCD population, neglecting distinct subtypes like contamination OCD. This oversight may contribute to the observed inconsistencies, emphasizing the need to account for specific OCD subtypes when analyzing ERPs. In this vein, the present study was conducted to identify the emotional components of ERPs associated with contamination-type OCD. Results showed differences in the patterns of ERP components between individuals with OCD and the healthy control group when viewing contamination-related pictures and neutral cues. In the frontal vertex (Fz), early and rapid components such as P3 and N2 were found to be significant. Then, in the Fz electrode, the N2 component only produced a greater negative amplitude in response to contamination-related pictures compared to neutral pictures in the OCD group, a finding consistent with the results of Ruchsow et al. (2007).

Processing of stimulus contrast is associated with the N2 ERP component (Folstein and Van Petten, 2008). N2 is characterized by a central frontal topography and is assumed to share a common neural substrate with the mid-cingulate cortex (Iannaccone et al., 2015). Additionally, this component is suggested to indicate the need for increased cognitive control to prevent response inhibition failure (Ullsperger et al., 2014; Iannaccone et al., 2015). While some studies showed an increase in N2 amplitude in OCD, others report evidence of a decrease or no difference in N2 amplitude (Keskin-Ergen et al., 2014). For instance, Ciesielski et al. (2011) demonstrated enhanced conflict monitoring in OCD during stimulus processing, reflected by larger N2 amplitudes. Leue and colleagues suggest that the N2 amplitude, as measured in the context of the study, was not affected by aversive reinforcement. Therefore, the domain of the N2 ERP component, in this particular study, does not show an increase or decrease in relation to aversive reinforcement (Leue et al., 2012, 2014). Similarly, a study examining patients with generalized anxiety disorder (Larson et al., 2013), a disorder that shares key symptoms with OCD such as excessive worry and threat estimation, found evidence for reduced N2 amplitude and compatibility with conflict in N2. Another pioneering study explored the emotional modulation of the N2 component and its relationship with attention interference in individuals with anxiety. The results of this study revealed that a decrease in N2 amplitude was linked to improved attention performance, particularly in the context of threat bias (Dennis and Chen, 2009). Investigating the consecutive modulation of experiments in conflict monitoring allows us to examine the observed hyperactivity in conflict monitoring in OCD further. There is some evidence that individuals with OCD show alterations in emotional processing, with increased sensitivity to negative emotional stimuli and difficulty in regulating emotional responses (Folstein et al., 2008; Gruner and Pittenger, 2017; Vaghi et al., 2017). It appears that patients with OCD are more sensitive to new conflicts compared to healthy participants. They also demonstrate overactive monitoring of efficacy or inefficacy in non-conflict situations where there is no demand for greater cognitive control. This may be perceived as an unnecessary effort, but it can also be associated with symptoms of agitation and doubt (Klawohn et al., 2014), or a high need to prevent potential harm. Therefore, in contamination OCD patients, the N2 component appears to be influenced by emotional processing, it can be concluded that these patients show high emotional sensitivity and hypervigilancy when facing opposite stimuli and increase the negative component of N2.

Furthermore, our results showed that only in the OCD group and in the P3 component of the Fz electrode the amplitude of the contamination pictures created a larger positive amplitude than neutral pictures, a result in line with findings of prior research (Ischebeck et al., 2011; Andreou et al., 2013). P3 is a positive component of event-related potential (ERP) that peaks after a stimulus for 300 milliseconds or more (up to 900 milliseconds). Unlike some of the previously evoked potentials, it is believed to be an “inward” component, meaning that it is highly dependent on the processing of the stimulus background, attention levels, and emotional arousal. It is interesting to note that P3 responses are observed when regular stimulus trains are interrupted by removing a stimulus or an unrelated stimulus, emphasizing the endogenous nature of this component. P3 component activity is mainly associated with a fixed pattern of generators with goal-related responses in the parietal and singular cortices and recent activation primarily in the prefrontal and subcortical parietal regions (Morand-Beaulieu et al., 2016). Larger P3 amplitudes were previously demonstrated in OCD patients compared to healthy control group participants in an auditory discrimination task, indicating faster cognitive processes, heightened arousal, and incorrect allocation of attentional resources (Gohle et al., 2008). Another study found that OCD patients had significantly higher P3 amplitudes than the healthy control group. The authors suggested that this increase in P3 amplitude may be related to alterations in cognitive processing in OCD, particularly in the context of attention and stimulus evaluation. Increased P3 amplitude in OCD patients may suggest that they are allocating more attentional resources to certain stimuli, which may contribute to the excessive repetitive behaviors and intrusive thoughts characteristic of the disorder (Chang et al., 2013; Raggi et al., 2021). Some studies investigated the brain response to cholecystokinin-tetrapeptide (CCK-4), a compound that induces panic attacks in susceptible individuals, in patients with OCD and healthy control groups. The study found that OCD patients had significantly higher P3 amplitudes in response to CCK-4 than the healthy control group. This may contribute to the anxiety and avoidance behaviors that are characteristic of OCD (Bogetto et al., 2000).

Nevertheless, there are also contradictory findings in the literature about the rule of the P3 component of ERP in OCD patients. In one study, findings indicated that individuals with OCD had significantly smaller P3 amplitudes compared to the healthy control group. The study also found that the P3 amplitude was negatively correlated with OCD symptom severity, suggesting that the smaller P3 amplitudes may be related to the cognitive deficits seen in OCD (Towey et al., 1994). For instance, some studies using visual methods have shown a reduction in the P3 domain in patients with OCD (although this was not the case in the present study), although patients in these older studies were under medical treatment (Malloy et al., 1989). Nonetheless, changes in P3 have been demonstrated in OCD patients compared to healthy individuals in research on auditory and visual domains (Gohle et al., 2008). Changes in P3 in non-pharmacological OCD patients appeared more as an increase in the domain. In contrast, in other psychiatric patients (such as schizophrenia or depression) that often show a decrease in this component, an increase in the domain was not observed (Dayan-Riva et al., 2019). In a study, Delplanque et al. (2004) utilized three distinct sets of pictures (unpleasant, neutral, and pleasant) to manipulate emotional valence in an oddball task paradigm. The manipulation of emotional valence resulted in differential effects on the P3 amplitude, with unpleasant pictures reducing P3 amplitude relative to pleasant pictures. Interestingly, these findings differ from the results of the current study. The observed effects on P3 suggest that this component is critical in modulating cognitive and emotional processing capacity in response to external stimuli. Therefore, as the results of the present study showed, the brain’s P3 component performance in individuals with OCD is different from that of healthy individuals, and this difference is in the frontal vertex (Fz). The most important finding regarding the roles of emotional components of event-related potentials (ERPs) in OCD is the altered processing of emotional stimuli compared to healthy control. Research studies have consistently demonstrated that individuals with OCD exhibit abnormalities in the neural processing of emotional stimuli, as reflected in their ERP responses. Specifically, these findings suggest that individuals with OCD show heightened neural sensitivity and increased emotional reactivity to stimuli associated with their obsessive concerns (Thomas et al., 2013). Moreover, studies have shown that individuals with OCD exhibit alterations in early ERP components such as the N2 and P3, which are associated with early sensory processing and attentional allocation to emotional stimuli. These abnormalities indicate disrupted processing of emotional information at the early stages of perception in individuals with OCD (Carlson, 2021). Furthermore, findings suggest that emotional ERP components, such as the P3 and the Late Positive Potential (LPP), are modulated by the severity and symptomatology of OCD. Greater symptom severity is associated with increased emotional reactivity and attentional bias towards OCD-relevant stimuli (Thorsen et al., 2018). Overall, these findings highlight the crucial role of the emotional components of ERPs in understanding the underlying neural mechanisms of OCD. They provide insights into the heightened emotional reactivity and attentional biases exhibited by individuals with OCD, shedding light on the emotional dysregulation that characterizes the disorder.

The present study has several limitations that need to be acknowledged. Firstly, the study sample consisted of a small number of patients specifically diagnosed with contamination OCD. The recruitment of OCD patients posed significant challenges, particularly due to the research being conducted during the peak of the COVID-19 pandemic. The restrictions and safety concerns associated with the pandemic impacted the ability to access and recruit a larger sample. Secondly, the present research utilized a passive visual task that specifically concentrated on stages of processing prior to response preparatory processes. This approach did not involve active, extroverted, or motor response demands. While this design choice allowed for a clearer and more focused examination of these stages, it is essential to recognize that the results obtained under laboratory conditions may not perfectly align with real-life situations. The absence of active engagement and motor responses in the task could potentially limit the generalizability of the findings to real-world contexts.

## Conclusion

Given the sensitivity of N2 and P3 components to emotional and cognitive impairments in OCD patients, particularly those with the contamination subtype, these components can serve as valuable guides for therapists. Our study highlights the potential of these event-related potential (ERP) markers as useful tools in therapeutic interventions. The pronounced manifestation of N2 and P3 components offers insights that can be leveraged to enhance the treatment process for individuals with OCD, leading to more effective outcomes and improved quality of life. While our findings briefly touched upon the potential for targeted treatments based on the neural markers identified in our study, we acknowledge the importance of providing more specific examples and practical suggestions for their implementation in clinical practice.

One promising approach is the use of LORETA (Low-Resolution Electromagnetic Tomography) neurofeedback. LORETA neurofeedback is a technique that allows for the assessment and training of brain activity in specific regions or networks in real-time. It provides information about brain activity patterns associated with various mental and emotional states. Although LORETA neurofeedback has been applied to various mental health conditions, its application in the treatment of OCD, particularly the contamination subtype, is an emerging and promising area of research (Ghasemzadeh et al., 2018). In addition, therapists can consider integrating emotion-based therapies, such as Emotion-Focused Therapy (EFT), that specifically target emotional processing deficits associated with the contamination subtype. These therapies build upon established first-line treatments for OCD and focus on enhancing emotional awareness, regulation, and processing. By incorporating these interventions that target both neural markers and emotional processing deficits, therapists can tailor treatment approaches for individuals with OCD, particularly those with the contamination subtype. This comprehensive approach may lead to more personalized and effective interventions, ultimately improving the therapeutic outcomes and overall well-being of individuals with OCD (Asmari et al., 2022).

## Data availability statement

The raw data supporting the conclusions of this article will be made available by the authors, without undue reservation.

## Ethics statement

The study procedures adhered to the ethical guidelines of the University of Social Welfare and Rehabilitation Sciences and were approved by the university’s ethics committee (Code Number = IR.USWR.REC.1398.018). The studies were conducted in accordance with the local legislation and institutional requirements. The participants provided their written informed consent to participate in this study.

## Author contributions

SSe: gathered the data, performed data analyses, and prepared the manuscript. BD, MN, SSh, and SSa: supervised the study and reviewed and revised the manuscript. All authors contributed to the article and approved the submitted version.

## Footnotes

^1^Mean difference.
